# Overweight and *Helicobacter pylori* infection: a correlation in metabolic dysfunction-associated fatty liver disease

**DOI:** 10.3389/fcimb.2025.1565298

**Published:** 2025-06-18

**Authors:** Xu Chen, Jiayue Fu, Kejia Jin, Zixuan Yang, Yidan Qian, Kehan Mei, Yihan Wang, Jialei Min, Yilin Du, Zaisheng Zhu, Shengcun Li

**Affiliations:** ^1^ Department of Medical Care Center, The First Affiliated Hospital of Wenzhou Medical University, Wenzhou, China; ^2^ School of Information and Engineering, Wenzhou Medical University, Wenzhou, China; ^3^ Oujiang Laboratory (Zhejiang Lab for Regenerative Medicine, Vision and Brain Health), School of Pharmaceutical Science, Wenzhou Medical University, Wenzhou, China; ^4^ Department of General Medicine, The First Affiliated Hospital of Wenzhou Medical University, Wenzhou, China; ^5^ Rehabilitation Medicine Center, The Second Affiliated Hospital and Yuying Children's Hospital of Wenzhou Medical University, Wenzhou, China

**Keywords:** metabolic dysfunction-associated fatty liver disease (MAFLD), *Helicobacter pylori* (*H. pylori*), body mass index (BMI), overweight, lean

## Abstract

**Background:**

Metabolic dysfunction-associated fatty liver disease (MAFLD) has recently replaced nonalcoholic fatty liver disease (NAFLD) as a term that more accurately describes its pathogenesis. *Helicobacter pylori* (*H. pylori*), a bacterium that infects over half the world’s population, has been increasingly linked to various extragastric diseases. However, the impact of *H. pylori* on MAFLD in the Chinese population remains unexplored.

**Methods:**

A retrospective cross-sectional study was conducted, encompassing 5619 participants from the First Affiliated Hospital of Wenzhou Medical University, spanning from April 2016 to August 2017. Detection of *H. pylori* was achieved through the ^13^C urea breath test or gastric biopsies with histochemical staining. Fatty liver was primarily diagnosed via ultrasound and assessed alongside metabolic indicators to confirm MAFLD. Logistic regression was utilized to evaluate the association between *H. pylori* and MAFLD.

**Results:**

No significant correlation between *H. pylori* infection and MAFLD was found in the overall population through either univariate (OR=1.136, 95%CI 0.995-1.297, p=0.059) or multivariate logistic regression analysis (OR=1.036, 95%CI 0.877-1.224, p=0.675). However, subgroup analysis revealed a significant association in overweight individuals (BMI ≥ 23 kg/m^2^) within the MAFLD group (51.2% vs. 35.5%, p=0.002), a pattern not observed in the non-MAFLD group (47.7% vs. 45.4%, p=0.126). This association persisted after adjusting for confounders (OR=1.957, 95%CI 1.176-3.256, p=0.010).

**Conclusion:**

Overweight individuals with MAFLD have a higher prevalence of *H. pylori* infection than their lean counterparts. This suggests a detrimental cycle between overweight status and *H. pylori* infection in MAFLD patients, potentially exacerbating metabolic deterioration. Therefore, eradication of *H. pylori* infection may have positive implications for reducing the incidence rate of overweight MAFLD.

## Introduction

Metabolic-dysfunction-associated fatty liver disease (MAFLD), proposed in 2020 as an alternative to non-alcoholic fatty liver disease (NAFLD), is a prevalent chronic hepatic disease (Eslam et al., 2020a). MAFLD is perceived as a standalone disease related to known metabolic dysfunction and has a specific positive diagnosis. It represents a spectrum of liver disorders, ranging from steatosis and steatohepatitis to fibrosis, cirrhosis, and hepatocellular carcinoma. Globally, MAFLD affects approximately 13.48% to 31.79% of adults (Younossi et al., 2016; Yang et al., 2017; Qu et al., 2021; Lazarus et al., 2022), with its prevalence in China rising from 23.8% in 2001 to 32.9% in 2018 (Zhou et al., 2020). In morbidly obese individuals, prevalence may reach up to 90% (Doulberis et al., 2017a; Polyzos and Kountouras, 2019). MAFLD patients often exhibit higher rates of hypertriglyceridemia, diabetes, and hypertension, and are at greater risk for significant fibrosis (Lim et al., 2023). A large longitudinal cohort study in China showed that the definition of MAFLD is more effective in identifying individuals with poor clinical features and poor prognosis than NAFLD (Xu et al., 2023). Especially for individuals with normal weight, the presence of steatosis with at least two metabolic risk abnormalities can also be diagnosed as criteria for MAFLD in non-overweight/obese subjects. Hence, the proposed standards would cover the full spectrum of phenotypes from metabolically unhealthy normal weight to metabolically unhealthy obesity.

*Helicobacter pylori* (*H. pylori*) is a Gram-negative spiral-shaped pathogen discovered by Marshall and Warren in 1983 from gastric mucosa (Marshall and Warren, 1984). It is estimated that approximately half of the global population is infected with *H. pylori* (Cover and Blaser, 2009), with rates in developing countries reaching up to 70% (Adenote et al., 2021). Chronic infection by *H. pylori* is recognized for its association with several gastrointestinal diseases, including chronic gastritis, peptic ulcers, gastric mucosa-associated lymphomas, and gastric cancer (Malfertheiner et al., 2012; Matsuhisa and Aftab, 2012). Recent studies have also demonstrated a growing correlation between *H. pylori* and various extragastric disorders, such as metabolic syndrome, insulin resistance (IR), type 2 diabetes mellitus (T2DM), cardiovascular diseases, and neurodegenerative diseases (Suzuki et al., 2006; Doulberis et al., 2017b; Gravina et al., 2020).

The correlation between *H. pylori* infection and liver disease has prompted extensive discussion, particularly after Cindomk et al. (Cindoruk et al., 2008) isolated *H. pylori* DNA from nonalcoholic steatohepatitis (NASH) liver tissue in 2008. Several high-quality meta-analyses suggest a positive correlation between NAFLD and *H. pylori* infection (Mantovani et al., 2019; Ning et al., 2019; Zhou et al., 2019; Heydari et al., 2022), potentially influencing metabolic risk factors, the gut microbiome, the inflammatory state, and other metabolically active hormones. However, studies in Chinese and European populations suggest that *H. pylori* may not be a risk factor for NAFLD (Cai et al., 2018; Fan et al., 2018; Wang et al., 2022).

Given the emerging concept of metabolic-dysfunction-associated fatty liver disease (MAFLD) and the significant role of overweight and obesity, as determined by body mass index (BMI), in diagnosing MAFLD, research has indicated an association between *H. pylori* infection and increased BMI and obesity risk (Chen et al., 2018). Following the eradication of *H. pylori*, patients have reported weight loss (Martin-Nunez et al., 2021). Therefore, we conducted a cross-sectional and cohort survey to investigate the relationship between *H. pylori* infection, MAFLD, and overweight.

## Subjects and methods

### Study population

This retrospective cohort study included individuals who underwent routine health check-ups, including liver imaging and either 13C-urea breath tests or gastroscopy biopsies, at the First Affiliated Hospital of Wenzhou Medical University between April 2016 and August 2017. Subjects were excluded if they were under 18 years old, had a history of cancer, major cerebrovascular accident, end-stage renal disease, gastrectomy, or chronic liver disease such as viral hepatitis or alcohol-induced liver disease, had used medications that could interfere with the accuracy of *H. pylori* testing within the specified time frames, such as proton pump inhibitors within two weeks or antibiotics, sucralfate, bismuth within one month, or had incomplete clinical information regarding *H. pylori* and fatty liver disease (Malfertheiner et al., 2017). The final analysis included 5325 participants: 4219 with MAFLD and 1106 healthy controls. The study received approval from the Ethics Committee of the First Affiliated Hospital of Wenzhou Medical University.

### Collection of clinical indicators

After obtaining informed consent, a structured questionnaire was administered to collect data on age, BMI, gender, medical history, medication use, smoking, and alcohol consumption. Participants were categorized as lean (BMI < 23 kg/m^2^) or overweight (BMI ≥ 23 kg/m^2^) (Eslam et al., 2020b). Smoking status was classified as current, former (quit for more than six months), or never, with the last two categorized as non-current smoking status. Alcohol consumption was classified as heavy (intake > 210 g per week for males and > 140 g per week for females) or non-heavy. Systolic and diastolic blood pressures were measured using an automated sphygmomanometer while seated. Blood biochemical parameters including fasting plasma glucose (FBG), hemoglobin A1c (HbAlc), alanine aminotransferase (ALT), aspartate aminotransferase (AST), alkaline phosphatase (ALP), gamma-glutamyltransferase (GGT), album, triglyceride (TG), total cholesterol (TC), high-density lipoprotein cholesterol (HDL-C), low-density lipoprotein cholesterol (LDL-C), uric acid, creatinine, serum calcium, serum phosphorus, 25 hydroxyvitamin D, high-sensitivity C-reactive protein (hs-CRP), red blood cell (RBC), hemoglobin and white blood cell (WBC) were assessed using an automatic biochemical analyzer.

### Diagnosis of *H. pylori* infection

*H. pylori* status was determined using a ^13^C urea breath test or gastric biopsies with histochemical staining (Malfertheiner et al., 2017). The ^13^C breath test involved: (a) collecting a baseline breath sample after a minimum of three hours of fasting; (b) administering ^13^C urea orally with warm water; (c) collecting a second breath sample 30 minutes later. A *H. pylori* infection was diagnosed if the Delta Over Baseline (DOB) value was ≥ 4. For gastric biopsies, the procedure included: (a) collecting mucosal specimens under endoscopic guidance by experienced physicians; (b) processing the specimens by fixing, dehydrating, embedding, slicing, and staining; (c) diagnosing via urease testing or histopathological examination.

### Definition of MAFLD

MAFLD is diagnosed by the presence of hepatic steatosis and one or more of the following conditions: (1) overweight or obese (BMI ≥ 23 kg/m^2^) (2) diabetes mellitus, or (3) at least 2 metabolic risk abnormalities. These abnormalities include: (a) waist circumference (WC) ≥ 90 cm for men and ≥ 80 cm for women, (b) blood pressure ≥ 130/85 mmHg or specific drug treatment, (3) fasting plasma triglycerides ≥ 150 mg/dl or on specific drug treatment, (4) plasma HDL-C < 40 mg/dl for men and < 50 mg/dl for women or specific drug treatment, (5) prediabetes (fasting glucose 100–125 mg/dl or hemoglobin A1c 5.7–6.4%, (6) homeostasis model assessment of insulin resistance score ≥ 2.5, (7) plasma hs-CRP level > 2 mg/L(1). lean MAFLD is defined as hepatic steatosis with a BMI <25 kg/m^2^ (or <23 kg/m^2^ in Asians) (Eslam et al., 2020b).

### Statistical analysis

Statistical analyses were conducted using SPSS 25.0 (SPSS Inc., Chicago, IL, USA). A P-value ≤ 0.05 was deemed statistically significant. Continuous variables were presented as means ± SD (for normally distributed data) or medians with interquartile ranges (for non-normally distribution date). Normality was assessed using the Kolmogorov-Smirnov test and normal Q-Q plots. The T-test was applied to normally distributed data, and the Mann–Whitney U-test was used otherwise. Categorical variables were expressed as counts and percentages and analyzed using the chi-square test or Fisher's exact test. Participants were stratified by the presence of MAFLD and/or *H. pylori* infection, as well as BMI. Multivariate logistic regression, adjusted for confounders, was employed to explore the relationship between groups, yielding odds ratios (OR) and 95% confidence intervals (CI).

## Result

### Baseline characteristics

This study included 5619 participants: 4461 (79.4%) non-MAFLD and 1158 (20.6%) MAFLD. Among these, 5325 underwent routine health screening for *H. pylori*; 2816 (52.89%) tested negative, and 2509 (47.12%) positive. Demographically, the MAFLD group was older (46.00 ± 10.95 vs. 47.37 ± 10.73, p<0.001), had a higher proportion of males (78% vs. 54.9%, p<0.001), and exhibited more obesity (BMI 26.11 ± 2.87 vs. 22.88 ± 2.95, p<0.001). The MAFLD group also had higher incidences of smoking, heavy drinking, and metabolic abnormalities such as hypertension, diabetes mellitus, hyperuricemia, and elevated liver enzymes (ALT, AST, ALP, GGT), as well as worse lipid profiles (higher triglycerides and lower HDL-C) (**Table 1**) (all p<0.001). However, the distribution of MAFLD relative to *H. pylori* infection showed no significant difference (49.6% vs. 46.5%, p=0.059).

**Table 1:** Baseline characteristics of all populations.

Variables	Total (n=5619)	non-MAFLD (n=4461, 79.4%)	MAFLD (n=1158, 20.6%)	p value
**Age, year**	46.28 ± 10.92	46.00 ± 10.95	47.37 ± 10.73	**<0.001**
**Male, n (%)**	3350 (59.6)	2447 (54.9)	903 (78.0)	**<0.001**
**BMI, kg/m^2^**	23.55 ± 3.21	22.88 ± 2.95	26.11 ± 2.87	**<0.001**
**Central Obesity, n (%)**	1443 (25.8)	825 (18.6)	618 (53.6)	**<0.001**
**Smoking, n (%)**	1616 (28.8)	1200 (26.9)	416 (35.9)	**<0.001**
**Heavy drink, n (%)**	1403 (27.9)	1048 (26.2)	355 (34.6)	**<0.001**
**Hypertension, n (%)**	1442 (25.7)	1016 (22.8)	426 (36.8)	**<0.001**
** SBP, mmHg**	124.14 ± 18.70	122.39 ± 18.52	130.79 ± 17.86	**<0.001**
** DBP, mmHg**	73.21 ± 12.81	71.88 ± 12.57	78.26 ± 12.46	**<0.001**
**DM, n (%)**	341 (6.1)	211 (4.7)	130 (11.2)	**<0.001**
**FBG, mmol/L**	4.98 ± 1.32	4.87 ± 1.17	5.40 ± 1.71	**<0.001**
**HbA1c, %**	5.59 ± 1.48	5.53 ± 1.55	5.85 ± 1.14	**<0.001**
**ALT, U/L**	22.00 (15.00,34.00)	20.00 (14.00,29.00)	35.00 (24.00,51.75)	**<0.001**
**AST, U/L**	24.00 (19.00,29.00)	23.00 (19.00,28.00)	28.00 (23.00,36.00)	**<0.001**
**ALP, U/L**	71.00 (59.00,85.00)	70.00 (57.00,84.00)	76.00 (64.00,93.00)	**<0.001**
**GGT, U/L**	28.00 (17.00,49.50)	24.00 (16.00,42.00)	47.00 (31.00,76.00)	**<0.001**
**TC, mmol/L**	5.22 ± 1.06	5.17 ± 1.05	5.43 ± 1.07	**<0.001**
**HDL-C, mmol/L**	1.28 ± 0.33	1.32 ± 0.34	1.11 ± 0.25	**<0.001**
**LDL-C, mmol/L**	3.09 ± 0.85	3.07 ± 0.85	3.20 ± 0.87	**<0.001**
**TG, mmol/L**	1.45 (0.99,2.22)	1.29 (0.93,1.90)	2.25 (1.61,3.11)	**<0.001**
**Uric Acid, μmol/L**	344.93 ± 91.16	332.47 ± 86.52	392.99 ± 92.68	**<0.001**
**Hyperuricemia, n (%)**	1344 (24.1)	861 (16.5)	483 (42.0)	**<0.001**
**Creatinine, μmol/L**	67.43 ± 14.58	66.53 ± 14.64	70.91 ± 13.79	**<0.001**
**eGFR**	108.37 ± 36.30	107.59 ± 17.54	111.06 ± 69.41	0.222
**Serum calcium, mmol/L**	2.31 (2.25,2.38)	2.31 (2.24,2.37)	2.33 (2.27,2.39)	**<0.001**
**Serum phosphorus, mmol/L**	1.07 (0.97,1.19)	1.08 (0.97,1.20)	1.06 (0.96,1.17)	**0.016**
**25 hydroxyvitamin D, nmol/L**	59.59 ± 21.18	59.51 ± 21.29	59.89 ± 20.76	0.613
**hs-CRP, mg/L**	0.73 (0.38,1.52)	0.64 (0.34,1.31)	1.13 (0.65,2.30)	**<0.001**
**RBC,** ×**10^^9^/L**	4.81 (4.48,5.15)	4.74 (4.43,5.07)	5.06 (4.75,5.33)	**<0.001**
**Hb, g/L**	144.95 ± 23.01	143.00 ± 24.47	152.45 ± 13.93	**<0.001**
**WBC,** ×**10^^9^/L**	5.86 (4.94,6.95)	5.71 (4.84,6.80)	6.41 (5.50,7.50)	**<0.001**
***H. pylori* infection**				0.059
** negative, n (%)**	2816 (52.9)	2259 (53.5)	557 (50.4)	
** positive, n (%)**	2509 (47.1)	1960 (46.5)	549 (49.6)	

MAFLD, Metabolic dysfunction-associated fatty liver disease; BMI, body mass index=weight (kg)/height^2^ (m^2^); SBP, systolic blood pressure; DBP, diastolic blood pressure. DM, diabetes; FBG, fasting blood glucose; HbA1c, glycated hemoglobin A1c; ALT, Alanine Aminotransferase; AST, Aspartate Aminotransferase; ALP, Alkaline Phosphatase; GGT, g-glutamyl Transferase; TC, Total Cholesterol; TG, Triglycerides; HDL-C, High-Density Lipoprotein Cholesterol; LDL-C, Low-Density Lipoprotein Cholesterol; hs-CRP, hypersensitive C-reactive protein; WBC, White Blood Cells; RBC, Red Blood Cells, respectively. Hb, Hemoglobin. Bold values indicated statistical significance P < 0.05.

### Logistic regression analyses for different markers and MAFLD

As displayed in **Table 2**, both univariate and multivariate logistic regression analyses were conducted to identify risk factors. Age, gender, BMI, smoking, heavy drinking, metabolic abnormalities, and hemoglobin were significantly associated with outcomes in the crude analysis (p <0.05). After adjustment, Age, BMI, smoking, FBG, ALT, HDL-C, LDL-C, TG, Uric Acid, and creatinine remained statistically significant (p<0.05). Gender, smoking, heavy drinking, hemoglobin A1c (HbA1c), and certain liver enzyme markers (AST, ALP, GGT) were no longer significantly correlated in the adjusted analysis. However, no significant relationship was found between *H. pylori* infection and MAFLD in the univariate analysis (p=0.059), nor after adjusting for confounding factors (p=0.675).

**Table 2:** Univariate and multivariate analysis of various markers in relation to MAFLD.

Variables	Crude OR (95% CI)	p value	Adjusted OR (95% CI)	p value
**Age, year**	1.012 (1.005 **-** 1.018)	**<0.001**	1.013 (1.004 **-** 1.022)	**0.007**
**Gender** ** (female vs. male)**	0.347 (0.297 **-** 0.405)	**<0.001**	0.866 (0.632 **-** 1.186)	0.369
**BMI, kg/m^2^**	1.437 (1.395 **-** 1.476)	**<0.001**	1.293 (1.252 **-** 1.335)	**<0.001**
**Smoking** ** (yes vs. no)**	1.481 (1.287 **-** 1.703)	**<0.001**	1.400 (1.149 **-** 1.706)	**0.001**
**Heavy drink** ** (yes vs. no)**	1.461 (1.258 **-** 1.697)	**<0.001**	1.068 (0.879 **-** 1.296)	0.509
**Hypertension** ** (yes vs. no)**	1.974 (1.713 **-** 2.274)	**<0.001**	0.838 (0.649 **-** 1.081)	0.173
**SBP, mmHg**	1.024 (1.020 **-** 1.028)	**<0.001**	1.008 (0.999 **-** 1.016)	0.072
**DBP, mmHg**	1.040 (1.034 **-** 1.045)	**<0.001**	1.007 (0.996 **-** 1.018)	0.203
**FBG, mmol/L**	1.286 (1.228 **-** 1.346)	**<0.001**	1.150 (1.077 **-** 1.228)	**<0.001**
**HbA1c, %**	1.234 (1.115 **-** 1.318)	**<0.001**	0.983 (0.928 **-** 1.042)	0.568
**ALT, U/L**	1.023 (1.020 **-** 1.026)	**<0.001**	1.010 (1.004 **-** 1.015)	**<0.001**
**AST, U/L**	1.018 (1.014 **-** 1.023)	**<0.001**	0.993 (0.983 **-** 1.002)	0.131
**ALP, U/L**	1.007 (1.004 **-** 1.010)	**<0.001**	1.001 (0.999 **-** 1.004)	0.335
**GGT, U/L**	1.005 (1.004 **-** 1.006)	**<0.001**	1.001 (1.000 **-** 1.002)	0.112
**TC, mmol/L**	1.265 (1.189 **-** 1.345)	**<0.001**	0.703 (0.549 **-** 0.900)	**0.005**
**HDL - C, mmol/L**	0.091 (0.070 **-** 0.120)	**<0.001**	0.446 (0.292 **-** 0.683)	**<0.001**
**LDL - C, mmol/L**	1.222 (1.131 **-** 1.319)	**<0.001**	1.748 (1.319 **-** 2.316)	**<0.001**
**TG, mmol/L**	1.721 (1.626 **-** 1.822)	**<0.001**	1.374 (1.235 **-** 1.528)	**<0.001**
**Uric Acid, μmol/L**	1.007 (1.006 **-** 1.008)	**<0.001**	1.003 (1.001 **-** 1.004)	**<0.001**
**Creatinine, μmol/L**	1.019 (1.015 **-** 1.024)	**<0.001**	0.992 (0.984 **-** 1.000)	**0.047**
**WBC,** ×**10^^9^/L**	1.004 (0.998 **-** 1.010)	0.208		
**RBC,** ×**10^^9^/L**	1.004 (0.995 **-** 1.014)	0.395		
**Hb, g/L**	1.036 (1.031 **-** 1.041)	**<0.001**	1.007 (1.000 **-** 1.014)	0.055
**Serum calcium, mmol/L**	1.003 (0.993 **-** 1.013)	0.555		
**Serum phosphorus, mmol/L**	1.004 (0.986 **-** 1.023)	0.665		
**25 hydroxyvitamin D, nmol/L**	1.001 (0.998 **-** 1.004)	0.613		
***H. pylori* infection (yes vs. no)**	1.136 (0.995 **-** 1.297)	0.059	1.036 (0.877 **-** 1.224)	0.675

OR, odds ratio; CI, confidence interval. Bold values indicated statistical significance P < 0.05.

### Tendency of *H. pylori* infection rate in different populations

**Figure 1:**
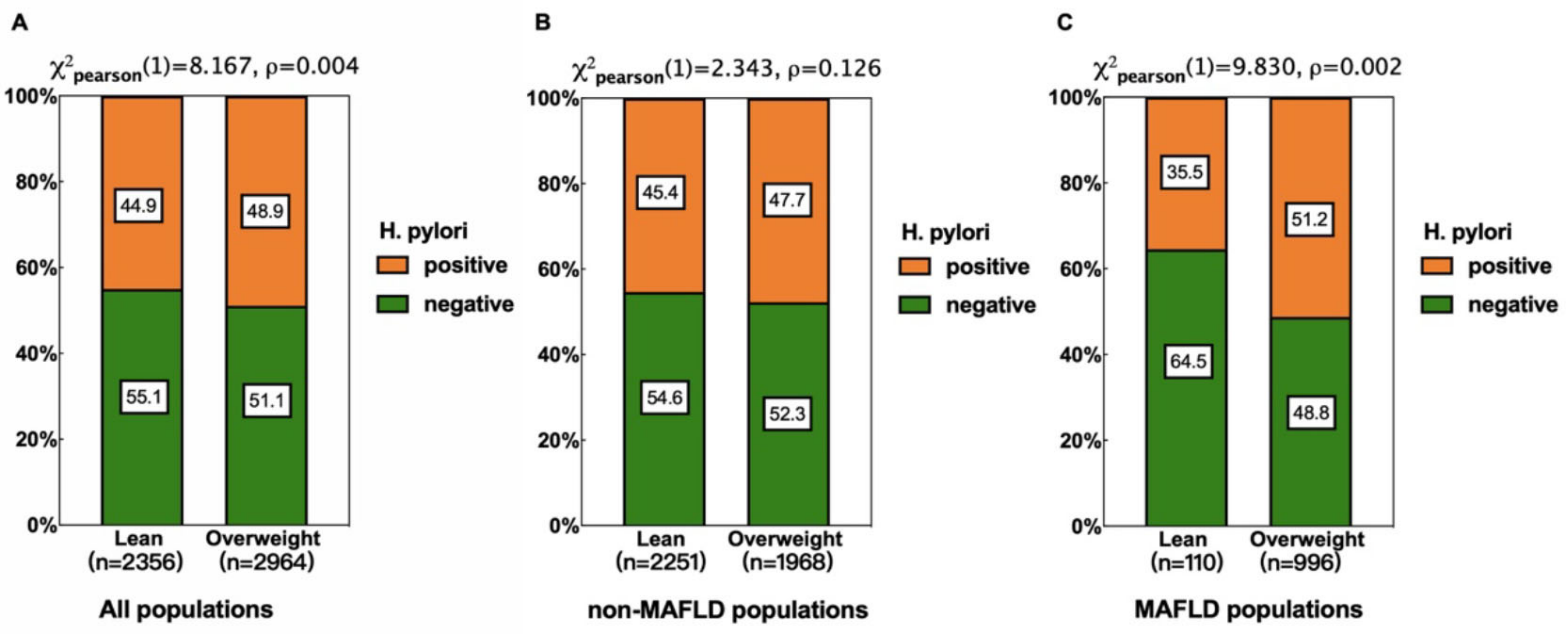
Relationship between *H pylori* infection and different weight in different populations. **(A)** All populations. **(B)** non-MAFLD populations. **(C)** MAFLD populations. Subgroup analyses revealed a higher *H. pylori* infection rate in overweight individuals in all populations **(A)**, with a more pronounced difference in the MAFLD subgroup **(B)**, but the differences were not significant in non-MAFLD populations **(C)**.

Based on body mass index, participants were categorized into two groups: lean (BMI<23 kg/m^2^) and overweight (BMI ≥ 23 kg/m^2^). Subgroup analyses of general, non-MAFLD, and MAFLD populations were performed using the chi-square test to further investigate *H. pylori* infection status across different weight categories and MAFLD statuses (**Figure 1**). Overall, the infection rate was higher among overweight individuals (48.9% vs. 44.9%, p=0.0004). This trend was more pronounced in MAFLD populations (51.2% vs. 35.5%, p=0.002) but was not significant in non-MAFLD populations (47.7% vs. 45.4%, p=0.126).

### Risk factors for MAFLD between lean and overweight groups

The baseline characteristics of the MAFLD population were shown in **Table 3**. The overweight MAFLD group had a significantly higher BMI (26.55 ± 2.50 vs. 21.77 ± 1.16, p<0.001) and more cases of central obesity (58.6% vs. 6.4%, p<0.001). Compared to the lean MAFLD group, the overweight group was younger, predominantly male, and included more smokers, had lower serum calcium levels and ALP, but exhibited higher diastolic blood pressure (DBP), creatinine, ALT, AST, GGT, and rates of hyperuricemia, RBD, Hb, as well as *H. pylori* infection rates (51.2% vs. 35.5%, p=0.002).

**Table 3:** Baseline characteristics of lean MAFLD and overweight MAFLD populations.

Variables	lean MAFLD (n=110, 9.9%)	overweight MAFLD (n=996, 90.1%)	p value
**Age, year**	49.85 ± 11.88	47.20 ± 10.36	**0.012**
Gender			
**Male, n (%)**	67 (60.9)	802 (80.5)	**<0.001**
**Female, n (%)**	43 (39.1)	194 (19.5)	
**BMI, kg/m^2^**	21.77 ± 1.16	26.55 ± 2.50	**<0.001**
**Central Obesity, n (%)**	7 (6.4)	584 (58.6)	**<0.001**
**Smoking, n (%)**	29 (26.4)	372 (37.3)	**0.023**
**Heavy drink, n (%)**	34 (30.9)	308 (30.9)	0.759
**HTN, n (%)**	45 (40.9)	363 (36.4)	0.357
** SBP, mmHg**	129.59 ± 20.23	130.71 ± 17.42	0.523
** DBP, mmHg**	75.35 ± 12.26	78.56 ± 12.46	**0.010**
**DM, n (%)**	16 (14.5)	111 (11.1)	0.288
**FBG, mmol/L**	5.61 ± 2.08	5.38 ± 1.69	0.178
**HbA1c, %**	5.91 ± 1.27	5.85 ± 1.14	0.636
**Creatinine, μmol/L**	65.06 ± 13.29	71.44 ± 13.50	**<0.001**
**Hyperuricemia, n (%)**	27 (24.5)	434 (43.6)	**<0.001**
**ALT, U/L**	27.00 (21.00, 39.50)	36.00 (25.00, 52.00)	**<0.001**
**AST, U/L**	25.00 (21.00, 30.25)	28.0 (23.0, 36.0)	**0.001**
**ALP, U/L**	86.20 ± 26.46	79.85 ± 24.59	**0.011**
**GGT, U/L**	39.00 (24.00, 60.00)	47.50 (31.00, 76.00)	**0.004**
**TC, mmol/L**	5.34 ± 1.15	5.46 ± 1.06	0.291
**HDL-C, mmol/L**	1.12 ± 0.22	1.12 ± 0.25	0.702
**LDL-C, mmol/L**	3.12 ± 0.90	3.23 ± 0.86	0.201
**TG, mmol/L**	2.17 (1.63, 2.92)	2.25 (1.60, 3.10)	0.562
**TP (total protein)**	75.88 ± 4.68	75.48 ± 4.38	0.362
**Albumin, g/L**	45.44 ± 3.06	45.59 ± 2.81	0.588
**Serum calcium, mmol/L**	2.35 (2.29, 2.40)	2.33 (2.27, 2.39)	**0.034**
**Serum phosphorus, mmol/L**	1.08 (0.97, 1.21)	1.06 (0.96, 1.17)	0.219
**25 hydroxyvitamin D, nmol/L**	61.03 ± 25.03	59.82 ± 20.33	0.589
**RBC,** ×**10^^9^/L**	4.89 ± 0.49	5.07 ± 0.44	**<0.001**
**Hb, g/L**	146.56 ± 15.97	153.33 ± 13.29	**<0.001**
**WBC,** ×**10^^9^/L**	6.45 ± 1.59	6.62 ± 1.63	0.295
***H. pylori* infection**			**0.002**
** negative, n (%)**	71 (64.5)	486 (48.8)	
** positive, n (%)**	39 (35.5)	510 (51.2)	

lean MAFLD is defined as hepatic steatosis with a BMI < 23 kg/m^2^ in Asians; overweight MAFLD is defined as hepatic steatosis with a BMI ≥ 23 kg/m^2^ in Asians. Bold values indicated statistical significance P < 0.05.

### Effect of *H. pylori* infection on overweight MAFLD

As shown in **Table 4**, univariate analysis identified several risk factors for overweight MAFLD classification by BMI, including age, male gender, smoking, DBP, ALT, GGT, ALP, hyperuricemia, creatinine, RBC, Hb, serum calcium, and *H. pylori* infection (p<0.05).

**Table 4:** Univariate analysis of various markers in relation to overweight MAFLD.

Variables	Crude OR (95% CI)	p value
**Age, year**	0.977 (0.959 - 0.995)	**0.013**
**Gender (male vs. female)**	2.653 (1.754 - 4.013)	**<0.001**
**Smoking (yes vs. no)**	1.665 (1.069 - 2.594)	**0.024**
**Heavy drink (yes vs. no)**	1.070 (0.693 - 1.654)	0.759
**Hypertension (yes vs. no)**	0.828 (0.554 - 1.237)	0.358
**SBP**	1.004 (0.992 - 1.015)	0.531
**DBP**	1.022 (1.005 - 1.039)	**0.011**
**Diabetes (yes vs. no)**	0.737 (0.419 - 1.297)	0.290
**FBG, mmol/L**	0.935 (0.848 - 1.032)	0.181
**HbAlc, %**	0.962 (0.819 - 1.130)	0.635
**Abnormal serum liver enzymes (yes vs. no)**	2.163 (1.453 - 3.222)	**<0.001**
**ALT, U/L**	1.014 (1.004 - 1.024)	**0.006**
**AST, U/L**	0.998 (0.991 - 1.005)	0.519
**GGT, U/L**	0.998 (0.997 - 1.000)	**0.039**
**ALP, U/L**	0.992 (0.985 - 0.999)	**0.017**
**Hyperuricemia (yes vs. no)**	2.375 (1.510 - 3.734)	**<0.001**
**Creatinine, μmol/L**	1.036 (1.020 - 1.052)	**<0.001**
**Dyslipidemia (yes vs. no)**	0.804 (0.394 - 1.639)	0.548
**TC, mmol/L**	1.108 (0.916 - 1.339)	0.290
**HDL - C, mmol/L**	0.858 (0.391 - 1.881)	0.702
**LDL - C, mmol/L**	1.165 (0.922 - 1.472)	0.200
**TG, mmol/L**	0.989 (0.901 - 1.086)	0.814
**WBC,** ×**10^^9^/L**	1.070 (0.943 - 1.213)	0.294
**RBC,** ×**10^^9^/L**	2.384 (1.522 - 3.733)	**<0.001**
**Hb, g/L**	1.034 (1.020 - 1.048)	**<0.001**
**Serum calcium, mmol/L**	0.380 (0.147 - 0.988)	**0.047**
**Serum phosphorus, mmol/L**	0.414 (0.124 - 1.389)	0.154
**25 hydroxyvitamin D, nmol/L**	0.997 (0.987 - 1.007)	0.589
***H. pylori* infection (yes vs. no)**	1.910 (1.268 - 2.878)	**0.002**

Bold values indicated statistical significance P < 0.05.

**Figure 2:**
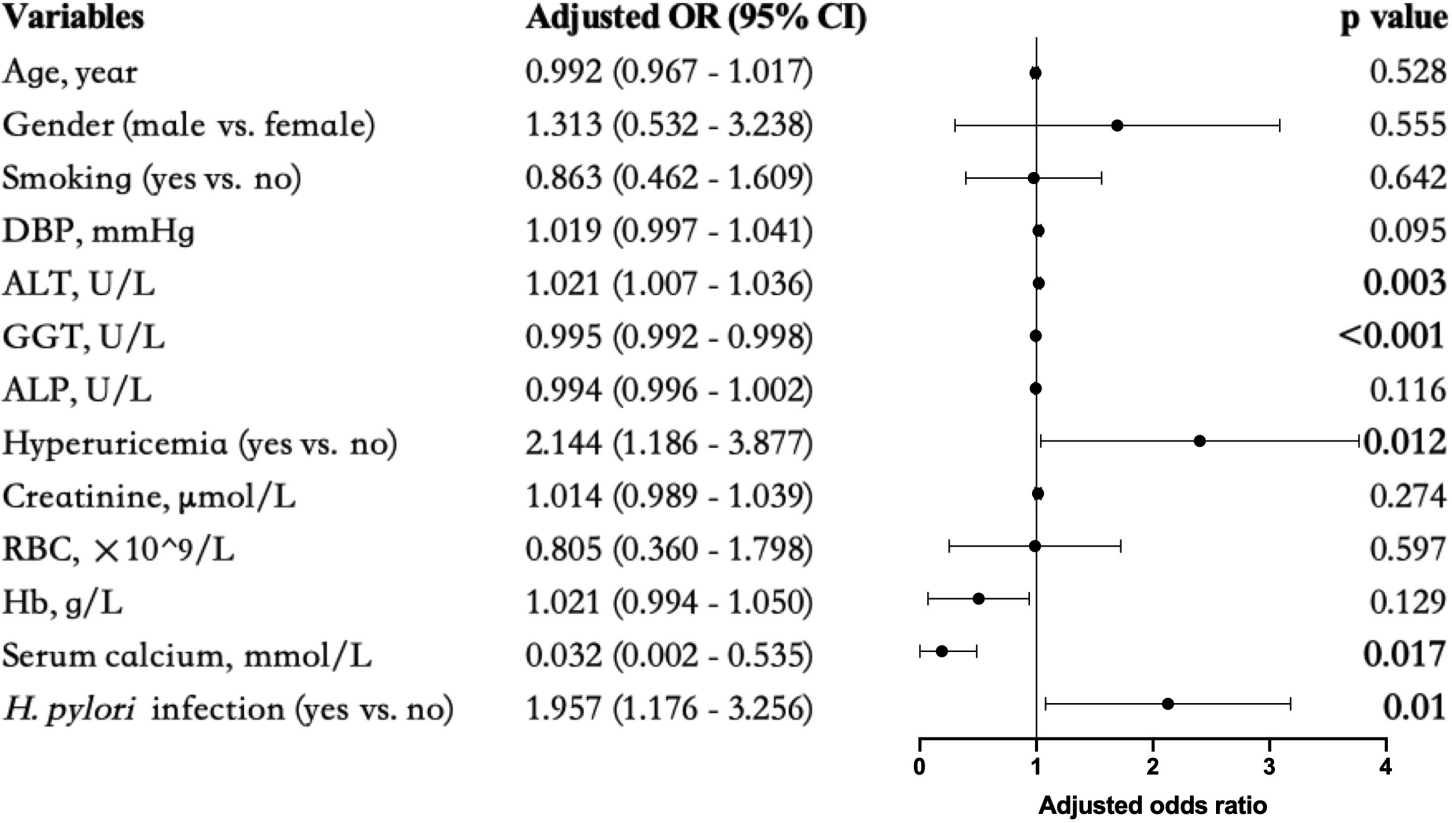
Forest plots of multivariate analysis for the association between different markers and overweight MAFLD. Bold values indicated statistical significance P < 0.05.

To control for confounding factors, adjustments were made in addition to age, gender, smoking (Model 0), DBP (Model 1), ALT, ALP, and GGT (Model 2), hyperuricemia and creatinine (Model 3), and RBC, Hb, and serum calcium (Model 4) respectively. The association between incident overweight MAFLD and *H. pylori* infection remained statistically significant (**Table 5**). To visualize this correlation, a forest plot was constructed based on Model 4 (**Figure 2**).

**Table 5:** Relationship between *H. pylori* infection and overweight MAFLD in different regression models.

Regression Models	Adjusted OR (95% CI)	p value
**Model 0**	1.915 (1.264 - 2.903)	**0.002**
**Model 1**	1.895 (1.249 - 2.875)	**0.003**
**Model 2**	1.998 (1.303 - 3.064)	**0.002**
**Model 3**	2.058 (1.337 - 3.165)	**0.001**
**Model 4**	1.957 (1.176 - 3.256)	**0.010**
**Model 0 is adjusted for age, gender, smoking.**		
**Model 1 is adjusted for Model 0 plus DBP.**		
**Model 2 is adjusted for Model 1 plus ALT, ALP, GGT.**		
**Model 3 is adjusted for Model 2 plus hyperuricemia, creatinine.**		
**Model 4 is adjusted for Model 3 plus RBC, Hb, Serum calcium.**		

Bold values indicated statistical significance P < 0.05.

## Discussion

The concept of MAFLD, introduced in 2020 as a replacement for Non-alcoholic Fatty Liver Disease (NAFLD), broadens the scope of fatty liver disease to include metabolic dysfunction as a central feature and has emerged as a significant global health concern (Eslam et al., 2020a). MAFLD increases the risk of end-stage liver disease, hepatocellular carcinoma, death, liver transplantation, and has extrahepatic consequences including cardiometabolic diseases and various cancers (Choi et al., 2022; Liu et al., 2022; Yuan et al., 2023; Crane et al., 2024; Fukunaga et al., 2024; Nakane et al., 2024). Its pathogenesis involves complex interactions among insulin resistance, adiposity, and lipid metabolism (Doulberis et al., 2017a; Polyzos and Kountouras, 2019; Lim et al., 2023; Xu et al., 2023).

In our study, we identified significant risk factors for MAFLD including older age, higher BMI, smoking, FBG, ALT, dyslipidemia (high TC, LDL-C, TG, and low HDL-C), uric acid, and creatinine. Further analysis revealed that lean MAFLD patients were older, had a higher percentage of females and hyperuricemia, and a lower proportion of smoking and drinking, lower diastolic blood pressure (DBP), and lower liver enzyme indicators (ALT, AST, ALP, and GGT) but higher serum calcium than overweight MAFLD patients. These results are consistent with previous studies on metabolic index changes and provide further insights into the risk factors for MAFLD (Eslam et al., 2020a; Chan et al., 2022; Yuan et al., 2022) and lean MAFLD (Buzova et al., 2020; Shah et al., 2020; Cheng et al., 2021; Eslam et al., 2022). Notably, we found a close correlation between high creatinine levels and MAFLD, persisting in further comparisons between overweight and lean MAFLD subgroups. A 2020 study (Sun et al., 2021) confirmed that the prevalence of CKD in MAFLD was higher than in non-metabolic dysfunction-associated NAFLD, and the severity of MAFLD was associated with a 1.34-fold higher risk of prevalent CKD. However, no significant differences were found in heavy drinking and hypertension between MAFLD and non-MAFLD. This discrepancy may necessitate further sample size expansion, and it is noted that fatty liver disease in Asia significantly differs from that in the West, where there are significantly higher rates of alcoholic liver disease (Younossi et al., 2022). Furthermore, when comparing the hypertension status of individuals aged 45 and above, those with MAFLD had a higher incidence of hypertension compared to those without MAFLD (44.9% vs. 32.4%, p<0.001). These findings suggest that individuals in the MAFLD group are more likely to possess metabolic risk factors and may require closer monitoring and management of their health conditions.

The scientific community has shown a growing interest in the relationship between MAFLD/NAFLD and *H. pylori*. However, it remains controversial whether *H. pylori* infection contributes to the increased risk of MAFLD. Several high-quality meta-analyses have demonstrated an increased risk of NAFLD in *H. pylori* -positive patients compared to *H. pylori*-negative patients, with odds ratios (ORs) ranging from 1.19 to 1.38 (Mantovani et al., 2019; Ning et al., 2019; Zhou et al., 2019; Heydari et al., 2022). Moreover, the eradication of *H. pylori* may reduce the risk of NAFLD (Polyzos et al., 2014). Although the majority of data supports a link between *H. pylori* infection and fatty liver disease, some studies dispute this association. Han et al. (2021) conducted a retrospective observational cohort study that showed no association between *H. pylori* seropositivity and NAFLD. This result is limited by the nature of *H. pylori* serology, which includes patients with past or present infection, and cannot accurately reflect active infection. Therefore, only active *H. pylori* infection should be considered when evaluating its relationship with NAFLD. Another study from southwestern China also found no positive correlation between *H. pylori* and NAFLD, but noted that *H. pylori* infection was more prevalent in patients with liver stiffness measurement (LSM) >7.4 kPa, suggesting an association with fibrosis (Liu et al., 2021).

Although no correlation between *H. pylori* infection and MAFLD was found in the overall population, a statistically significant correlation was observed between *H. pylori* infection and overweight in the MAFLD population, but not in the non-MAFLD population. Even after adjusting for multiple confounding factors in multivariate logistic regression, *H. pylori* infection remained an independent risk factor for overweight MAFLD patients compared to lean MAFLD. This suggests that *H. pylori* infection may synergistically promote the progression of overweight MAFLD. In other words, metabolic health is a dynamic state throughout the life cycle, and *H. pylori* infection may be a determining factor during the progression of overweight MAFLD phenotype. Previous research suggests that the pathophysiological mechanisms may involve *H. pylori* infection being associated with elevated levels of serum fetuin-A, which impairs insulin signaling via inhibition of insulin receptor tyrosine kinase activity and promotes inflammation through TLR4 activation (Pal et al., 2012; Goustin et al., 2013; Kahraman et al., 2013; von Loeffelholz et al., 2016). Additionally, *H. pylori* infection may affect gut barrier permeability, leading to the translocation of PAMPs to the liver via the gut-liver axis, thereby initiating inflammation and fibrosis (Quaresma et al., 2006; Dash et al., 2019). It is also linked to reduced serum levels of high-density lipoprotein (HDL) cholesterol, which promotes dyslipidemia (Upala et al., 2016). Moreover, there is an association between *H. pylori* infection and obesity. The infection induces inflammatory responses in gastric cells responsible for leptin and ghrelin production (Roper et al., 2008). Due to the anorexigenic effect of leptin, *H. pylori* infection may stimulate overeating and contribute to obesity mechanisms (Schwartz et al., 1996; Shintani et al., 2001). Obesity is typically associated with impaired immune function, and immune deterioration correlates with the degree of obesity (Marti et al., 2001). Studies have indicated that the maturation of monocytes into macrophages is diminished, and the bactericidal capacity of PMN cells is reduced in obese individuals (Palmblad et al., 1980; Krishnan et al., 1982), suggesting that the compromised immune state in obesity diminishes the ability to resist *H. pylori* infection.

For the first time, our study discovered that *H. pylori* was more prevalent in overweight MAFLD patients than lean individuals. However, the study has certain limitations that merit attention. Firstly, this is a single-center retrospective study, which may limit the generalizability of the findings. Multicenter, longitudinal studies are needed to provide more robust evidence. Secondly, because MAFLD is mainly diagnosed by ultrasound, it is not possible to determine the severity of MAFLD-associated hepatitis, and mild steatosis may go undetected. Nevertheless, the high sensitivity and specificity of ultrasound diagnosis for fatty hepatitis have led to its widespread use in clinical practice. Thirdly, we defined metabolic dysregulation as the presence of at least two of five metabolic risk abnormalities due to limited data on insulin resistance and high-sensitivity C-reactive protein, which may reduce the detection rate of MAFLD. Finally, although methods are being used to adjust for confounding factors, there may still be potential influences from other factors.

## Conclusion

This study suggests a clear relationship between *H. pylori* infection and overweight MAFLD, indicating a vicious cycle between the two in MAFLD patients, leading to a worsening of metabolic status. Therefore, addressing the weight issues and *H. pylori* infection status of MAFLD patients may be beneficial for disease assessment. Moreover, controlling *H. pylori* infection may be a modifiable risk factor for preventing or treating overweight MAFLD. Further studies are needed to determine whether eradicating *H. pylori* and controlling body weight can improve metabolic associated fatty liver and prevent further liver disease.

## Data Availability

The original contributions presented in the study are included in the article/supplementary material. Further inquiries can be directed to the corresponding authors.
